# Gold Inlays without Solder

**Published:** 1906-04

**Authors:** Alex. Jameson

**Affiliations:** Indianapolis, Ind.


### GOLD INLAYS WITHOUT SOLDER.

By Alex. Jameson, D. D. S., Indianapolis, Ind.

After preparing the cavity for an inlay, I obtain an impression in modeling compound. This is mounted in a rubber ring filled with soft plaster, in such a way that it will be the bottom of a cavity which is otherwise cone-shaped. Into this cavity I fill, either amalgam or achite cement, and allow to harden. After separation I have a model of the cavity in one of these substances. This model I mount in plaster or compound in the little steel ring which goes into my swager. Although it is not absolutely necessary I usually take a piece of pure gold, 33 or 36 gauge, and annealing several times make a matrix to approximately fit this model, finishing the matrix by means of the unvulcanized rubber and the pressure of a heavy hammer. If I have any doubt about the accuracy of my impression, I fill this matrix with hard wax and try into the original cavity, and just here allow me to say that if I have used archite this process has not taken so long that I could not keep my patient.

Presuming that the fit is perfect I trim all edges practically as far as necessary, remove, burn out the wax, and replace in the model. Now with any of the fibre golds, annealed as for filling, I press quite a large mass into the matrix (in place), and proceed to partially consolidate against the walls and bottom of cavity. The ring with model and matrix, etc., is now placed in the swager and a piece of tissue paper is placed over the gold; unvulcanized rubber is placed over the paper and the plunger after being put into position is driven down hard with the hammer. After taking the ring out the paper is removed, and the piece is annealed. After it has cooled (dry) it is replaced and the process continued until you have a homogeneous filling thoroughly condensed. The rest of the process is the simple one of placing in the cavity and trimming, polishing, etc. Where you have doubts as to condensation, or where you want a harder filling for masticating, you may, if you wish, add solder to the outside in such quantities as you may deem necessary.

I will say that I have shown inlays made in this way to other dentists and in some instances have had a hard time to convince them that they were not gold fillings, so accurately were they placed and so good was the color, being pure gold. I do not claim that there are many advantages in this process. I will say, however, that given a large cavity properly prepared I can take an accurate impression and complete and set the inlay in a much shorter time than I can fill the tooth or make an inlay in any other acceptable way.

A few words about the swager: It is one I had made at a machine shop, of steel, the dimensions are 7/8 long by 1 3/8 diameter outside. Inside diameter, 11/16. Plunger fits snugly and is one inch long. I have several steel and brass rings which slip inside and are about 1/4 inch high; in these I mount models, etc. This swager may have a special base, or may be simply placed on a heavy plane metal plate as a die plate.—*Items of Interest.*

